# Stacked PAP Flaps for Two-Stage Immediate Bilateral Breast Reconstruction: A Case Report

**Published:** 2017-05

**Authors:** Luis Parra

**Affiliations:** Department of Plastic Surgery, Hospital 12 de Octubre, Madrid, Spain

**Keywords:** Stacked flaps, Pap flap, Breast reconstruction

**DEAR EDITOR**

Although abdominal tissue remains the gold standard in autologous breast reconstruction,^1^ other donor sites like buttocks, thigh, dorsal area are often used. The posterior thigh flap was first described in 1980 by Hurwitz and posteriorly transferred as a free flap by Song *et al*. in 1984. Pap flap has gained popularity, as it offers a favorable response based on a consistent vascular anatomy, ease of harvest, and low morbidity profile,^2^ being considered, as an excellent alternative to the anterolateral thigh perforator flap for head and neck and limb reconstruction. Today, it is increasingly used for autologous breast reconstruction. The ideal patient for the latter has a breast of small to moderate size, with previous surgery or limited donor tissue on the abdominal area.^2^^,^^3^

A 57-year-old female with a right breast cancer, arrived to our unit in July 2015. Mastectomy and autologous reconstruction with a DIEP flap was the treatment suggested to our patient. At the same time, the contralateral breast was reduced to achieve symmetry with the reconstructed breast. On the histopathologic study of the left breast specimen a low grade intraductal carcinoma was evidenced. The patient was taken once again to the operating room to perform a mastectomy with an immediate reconstruction bearing in mind the family history and her request.

A large flap was still needed to fill the mastectomy defect, so bilateral pap flaps measuring 17×9 cm were designed and harvested based on a single perforator artery, found by preoperative Doppler sonography. The perforator of the right flap was positioned anterior in our design and intraoperatively its course was found to be completely septocutaneos. The perforator of the left flap, being positioned in a more posterior way, had a muscular course of 4,3 cm through the adductor magnus muscle. The mean elevation time was about 75 min and 95 min respectively. 

The recipient site was prepared removing the third costal cartilage as well as some of the intercostal muscles to expose the internal mammary vessels. Once raised, both flaps were transposed to the mastectomy site, the right pap flap was positioned on the superior half and anastomosed to the anterograde mammary vessels in an end to end fashion. The left pap flap was anastomosed to the retrograde limb of the recipient vessels in and end to end fashion and positioned on the inferior portion of the pocket. Arterial anastomosis was quite challenging due to the high discrepancy between the flap and the recipient vessels. Both flaps were widely de-epithelizedand sutured together, only a small skin paddle was left over the flap, positioned in the areola and the vertical limb of the reduction pattern to allow monitoring flap vitality.

There were no complications during the surgery, and the postoperative period was uneventful. The patient was discharged home on the fifth postoperative day, one of the tube drainage was removed on the 5^th^ day after the surgery, and the other one on the 8^th^ day. All the donor incisions healed well. We asked specifically about the postoperative discomfort, pain and first esthetical impressions, and to make a comparison between the two autologous reconstructions. The patient referred no pain at all, mild tightness sensation in the inner thigh region, but did not experience difﬁcultly in returning to normal activity*, *and was highly satisfied with the aesthetical results. Another surgical procedure will be offered to our patient for the reconstruction of the nipples and to equalize both breasts if desired. 

Robert Allen *et al.* described a series of 27 Pap flaps for breast reconstruction, all them were successful with minimal complications and minimal fat necrosis.^4^ In some situations, a large volumen of tissue is needed to achieve a good cosmesis and symmetry with the contralateral breast, combined flaps^5^ or performing two “stacked flaps” to be reliable and feasible choices.^6^

In our case, in spite of performing a breast reduction pattern, large flaps were still needed. Some articles described the increased donor site morbidity in abdominal free flaps with regards to the amount of fascia harvested^7^ or when bipedicled flaps were dissected.^8^ In our case, although unintentionally, three flaps were obtained and just one abdominal fascia incision. These findings could be of interest when needing large volumes, reducing the number of flaps needed thus saving operating time and reducing the risk of donor site complications.

Blechman *et al.* described the first stacked Pap flap for unilateral reconstruction in a patient with a Poland Syndrome^9^ and Stalder *et al.* described unilateral stacked profunda artery perforator flap reconstruction in five patients, one of them performed in an immediate fashion. They anastomosed the superior flap to the retrograde internal mammary system and the inferior flap to the antegrade internal mammary system.^10^

The retrograde internal mammary vessels have been widely described as good receptor vessels, focusing more on the vein as an alternative outflow for congestive flaps.^11^ However, some authors still consider that it is not reliable enough due to the findings describing the peak venous blood velocity on the retrograde limb resulted to be significantly slower than that of the antegrade vein.^12^ On the other side, limited number of reports are available describing the use of the retrograde internal mammary artery as a sole vessel, but clinical and experimental data support the concept that this system can be used without fear of compromising flap viability.^13^

We have used the internal mammary artery in a retrograde fashion several times but is the first time we use the retrograde vein as a single recipient vessel. In the absence of abdominal tissue, Pap flap remains a good alternative for breast reconstruction with a suitable pedicle and concealing scar. One large DIEP flap and two stacked pap flaps may be a reasonable solution to reconstruct large breasts.

## CONFLICT OF INTEREST

The authors declare no conflict of interest.
